# A global wildfire dataset for the analysis of fire regimes and fire behaviour

**DOI:** 10.1038/s41597-019-0312-2

**Published:** 2019-11-29

**Authors:** Tomàs Artés, Duarte Oom, Daniele de Rigo, Tracy Houston Durrant, Pieralberto Maianti, Giorgio Libertà, Jesús San-Miguel-Ayanz

**Affiliations:** 1 European Commission, Joint Research Centre (JRC), Ispra, Italy; 2 External consultant for the European Commission (ARCADIA SIT s.r.l) ARCADIA SIT s.r.l, Vigevano, PV Italy; 3 Maieutike Research Initiative, Milan, MI Italy; 4 External consultant for the European Commission (Engineering Ingegneria Informatica S.p.A.) Engineering Ingegneria Informatica S.p.A., Rome, Italy

**Keywords:** Natural variation in plants, Natural hazards

## Abstract

Global fire monitoring systems are crucial to study fire behaviour, fire regimes and their impact at the global scale. Although global fire products based on the use of Earth Observation satellites exist, most remote sensing products only partially cover the requirements for these analyses. These data do not provide information like fire size, fire spread speed, how fires may evolve and joint into single event, or the number of fire events for a given area. This high level of abstraction is very valuable; it makes it possible to characterize fires by types (either size, spread, behaviour, etc.). Here, we present and test a data mining work flow to create a global database of single fires that allows for the characterization of fire types and fire regimes worldwide. This work describes the data produced by a data mining process using MODIS burnt area product Collection 6 (MCD64A1). The entire product has been computed until the present and is available under the umbrella of the Global Wildfire Information System (GWIS).

## Abstract (summary)

**Table:** 

Measurement(s)	wildfire
Technology Type(s)	digital curation • computational modeling technique
Factor Type(s)	date of fire • geometry of burnt area
Sample Characteristic - Environment	agricultural field • forest ecosystem
Sample Characteristic - Location	Earth (planet)

Machine-accessible metadata file describing the reported data: 10.6084/m9.figshare.10284101

## Background & Summary

Wildfires are an important phenomenon at the global scale, as they are responsible for large amounts of economic and environmental damage. These effects are being exacerbated by the influence of climate change. Wildfire regimes are changing, wildfire seasons are becoming longer^1^, wildfire average sizes are increasing in many areas of the world and wildfires are happening in areas in which they did not occur in the past. Climate change is driving some forests into a stressed state^2^, reducing the vegetation water content and leading to very prone-fire landscapes where high-severity wildfires could occur. Recently, fire danger indicators such as the Canadian Fire Weather Index (FWI)^3^ have shown the highest values registered during the last 37 years in many European countries. High values of the drought code (DC) and the duff moisture code (DMC), which are components of the FWI, are some of the common factors in the large fires that have recently burned in many regions of the world.

When analysing the factors involved in fire danger, fire behaviour and overall fire effects, there is a considerable amount of uncertainty and missing data^4–6^. It is possible to collect information from the local authorities for a specific event; however it is not feasible to have the same approach for a global wildfire behaviour analysis. Global data and information about wildfires is fragmented and inconsistent in many areas of the world^7^. Global burned area products derived from satellite imagery provide information on spatial and temporal attributes of all areas affected by fires, but they do not contain information on single wildfire events^8^. This fact makes it impossible to distinguish certain wildfire types or study their behaviour or occurrence based on the dynamics of individual events.

In order to analyse wildfire regimes and the dynamics of wildfire events, a database at global scale describing single fire events and their properties becomes crucial^9^. Several recent studies provided accurate information on the individual fire number/size distribution^10–12^ or on fire spread rates^13,14^ of individual fire events at global/regional scale using burned area products. Recent publications describing wildfire events such as FRY^12^ or Fire Atlas^14^ focus more on the fire patch behavior than on the characterization of the fire events. Although the goals of Fire Atlas are similar to the objectives in out work, it differs in the methodology and the criteria used to describe the fire events.

Here, we present a global single wildfire database and the resulting data, the GlobFire Database, based on an algorithm that relies on encoding in a graph structure a space-time relationship among patches of burned areas. This approach, also used in other studies^15,16^, allows us to have wildfires with several initial ignition points and merge them even if burnt areas are non-spatially contiguous such as spotting fires. The method is independent from the tiling system of the sources, since the process is carried out globally and the identification of the fires is not performed inside a tile, which differs from the previous works. Besides, it is possible to use several burnt area products at the same time and combine them. It is possible to extract from the GlobFire database, the single wildfire events, their properties and their evolution. It is also be possible to search for general patterns of wildfires at diverse spatial scales (including global), providing critical information to fire spread and fire behaviour analysis, only available at local, regional or country level^17^. This information can also be valuable for fire regime characterization at global scale.

The newly developed database will add information to the current global burnt area products by developing the first uniform and single wildfire event database at global scale. This information will be ingested and fully available in the Global Wildfire Information System (GWIS) platform^18^, a joint initiative of the Group on Earth Observations (GEO) and Copernicus Work Programs. The main goal of GWIS is to gather and process existing data at regional and national level to provide a comprehensive view and evaluation of the fire regimes at global scale.

The database described in this work would allow users to focus on wildfires of special interest or to characterize environmental factors for different fire types by analysing fire behaviour.

In order to evaluate the algorithm and the database, the NASA Moderate-Resolution Imaging Spectroradiometer (MODIS) burnt area Collection 6 product (MCD64A1) has been chosen, although any similar burned area product could be added as input, and it is even possible to combine several sources during the data mining process. The proposed method works with raster or vector patch based burnt area products such as those derived from MODIS^19^, Copernicus Proba-V^20^ or Fire CCI^21^.

## Methods

Within GlobFire, a single wildfire event is considered as an abstract representation of a fire that can be stored in a database. An abstract representation is done with a small class diagram^15^ that is flexible enough to add new properties to the fire and which contains the minimum elements to describe a fire event. For instance, it makes it possible to store the initial and final date of the fire, spatial data describing the fire perimeter and its burnt area for every individual time step, and sources used for each iteration in the evolution of a fire. Each abstract representation of the fire is referred to a fire object.

Then, a collection of fire objects could describe the evolution of burnt areas for the entire globe and could be easily indexed by space and time, and finally stored in a spatial database. Currently, the source burnt area data are not produced in real time; the updates are released weekly or monthly. Assuming that, at some point, an accurate burnt area product may be produced in real time, the process should be able to deal with a continuous accumulative update. Its processing time should be less than the frequency between each update. The proposed work flow is able to keep the fire object collection with the potential active fires only and archive the ones that will not interact with the next updates. This allows us to keep a sustainable fire object collection of potential active fires and non-active fires. Besides, the method differs from that of the Fire Atlas because it allows us the use of different thresholds for different areas of the world, and the fire identification is independent from the tiling system used on the input data. It is a flexible method that can easily merge different resolution input sources and improves previous approaches to identify wildfire events, avoiding reliance on methods used for raster sources. The proposed method does not use tiles because it relies on the spatial indexes to optimize the memory usage, creating a compressed distance matrix by rows.

There are some thresholds that define the fire event based on the burnt activity. In this work, a fire event is considered as a set of burnt areas that are connected by touching or intersecting. Touching burnt areas are considered as different fires if there is a time distance between them of more than 5 days.

Finally, the fire is flagged as not active if no burnt areas have been added to it for 16 days and it is archived. One advantage of the method shown in this work is that the thresholds, 5 and 16 days in this work, could depend on the location of the fire. Therefore, if local organizations or authorities define their own thresholds, they can be included in the algorithm.

The data mining assumes a set of geometries as an input. Consequently, any burnt area product used should be transformed to vector format. This last fact implies known disadvantages; for example it becomes not feasible to keep quality attributes of each pixel, especially for high resolutions. Nonetheless, the main goal of this process is not to keep the entire data included in the sources but to mine data about individual fire events from different sources and resolutions. One advantage of the transformation to a vector format is that any set of touching pixels become a patch, an area that was burnt during the same time (daily based for MCD64A1).

**Fig. 1:**
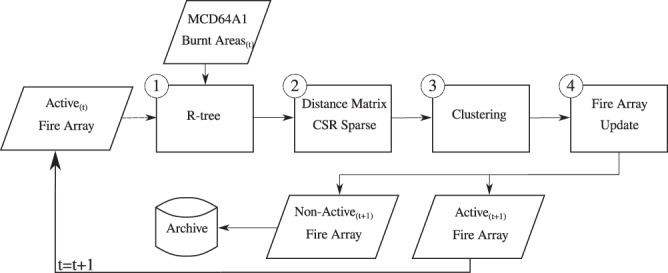
Diagram depicting the data mining process for each execution step. The first step is the creation of an R-tree with those fires that are considered active. Using the R-tree with the new burnt areas from MCD64A1 in vector format a distance sparse matrix compressed by rows is created considering the minimum distance between geometries and time stamps. Next, the compressed sparse matrix by rows is used as input for the DBSCAN, which will return the labels for the clusters. Finally, the fire array is updated with the labels, and those fires which are no longer considered as active are archived.

Figure 1 depicts the scheme of the data mining workflow. The process starts with those fires which are active and have been detected in a previous execution (*Active*_(*t*)_ *Fire Array*). These fires are introduced into an R-tree^22^ (Python ctypes wrapper (http://toblerity.org/rtree/) of libspatialindex (https://libspatialindex.github.io/) shown as step 0 in the Fig. 1) with the the new burnt pixels and pixel patches (*Burnt Areas*_(*t* + 1)_). This R-tree is different for each execution. It depends on the location of the fire objects of the previous iteration. These fire objects are instances of the class previously described^15^. Each object is a fire event. This step removes the need to use any tile system and allows us the use of several sources suitable to be transformed to a vector format instead previous works^14^. Afterwards, it is possible to build a compressed sparse matrix by rows (CSR) using the minimum distance between the burnt pixels of previous fires and new burnt pixel patches. The CSR matrix describes an all-to-all distance matrix, but removes such distances that are not relevant because they belong to different parts of the R-tree (depicted in Fig. 1 as step 1). For a given burnt pixel or patch, the R-tree provides those burnt elements with an intersection in its bounding box. At that point, the geometries with touching or intersecting bounding boxes can be used to compute a distance for the given pixel or burnt patch. The CSR matrix is used as a precomputed distance matrix for a later processing step which is a DBSCAN^23^ clustering using the implementation included in scikit-learn^24^. The DBSCAN will provide the labels for each cluster based on a distance matrix, distance threshold and a minimum number of elements of a single cluster. This step is shown in Fig. 1 as step 2.

The labels provided by the DBSCAN contain the information about that sets of pixels that belong to the same event. Hence, processing this information we update the fire event array. The labels provide updated information about the events. Therefore, processing the labels, new burnt areas are added to active fire events, new fire events are created and also active fire events are joined in a single one.

Finally, the fire array is divided in two lists, active or non-active (Fig. 1 step 3). The criterion for this classification is a threshold of 16 days. Those fires without a new burnt area added to them during the time threshold of 16 days are considered as non-active. Therefore, the non-active fires are not included in the next execution and are stored in the database with all their attributes and daily burnt areas. Reducing this threshold, the workload during the processing is notably reduced. However, because of the very diversified nature of fires at global scale (root fires, peat fires^25^, etc.) the threshold should be high enough to be used in different regions and take into account fires which last from a single day to months.

During the uploading process into the database, the geometries are simplified without information loss, only removing the points that are not required to describe the geometry. For instance, intermediate points inside the same straight line are removed. This process is done to avoid an increase of complexity and to reduce the amount of storage used for the database.

The method described carries out a spatio-temporal clustering. Commonly, wildfire burnt area product deliveries are aggregated weekly or monthly. Therefore, the method includes the time in the matrix distance. In cases where the pixel or patch are more than 5 days apart from each other they are considered as different events. However, this threshold can be ignored and defined to the maximum temporal difference available in each burnt area product delivery. The implicit iterative nature of the algorithm and the inner time stamp analysis allows us to work with date and time formats and to additionally work with other burnt area products and mixing sources.

The parameters used in DBSCAN to generate the labels for the event clustering are at least 1 element to create a new label and the spatial threshold of 0.01 degrees, which is constant for all the globe. Adding an extra step with another R-Tree and running the DBSCAN several times, the spatial and temporal thresholds could vary for different areas and time of the year. However, the criteria to define these thresholds for each time and for different locations often require local knowledge or a focus on fire types. The fire event identification can be applied hierarchically using different nested loops and labelling in detail fire complexes.

### Execution

This section describes the computational environment, the amount of data introduced into the GlobFire database and the inputs used during the execution of the algorithm developed for the method explained in the previous section. The section also shows the resources that have been used and depicts the amount of resources required to reproduce the execution of the method.

All the executions were carried out in a IBM System x3550 M4 with the following specifications:

- CPU: Intel(R) Xeon(R) CPU E5-2697 v2.
- RAM: 192 GB with 32 GB LRDIMMs.
- Storage: With IBM ServeRAID M5110, 3.3TB.
- OS: CentOS Linux release 7.5.1804 (Core) 3.10.0-693.5.2.el7.x86_64.

## Data Records

As mentioned in the Method section, the algorithm generates an array of fire objects. The data is published creating ESRI Shapefiles from those arrays containing the fire identification, the initial date, the final date, the geometry and a field specifying if it is a daily burnt area or a final burnt area. In addition, two dumps of a PostgreSQL GlobFire database are also provided. The first dump contains the final areas. Each row of the table (fire event) has an initial date, end date, multi-polygon geometry of the burnt area and a numerical identifier. This last identifier can be used as a key to link the second dump which contains the daily burnt areas for each fire. See Table 1.

**Table 1:** Description of the yearly tables and its fields for the PostgreSQL database.

Final Burnt Area		Daily Burnt Area	
Field	Type	Field	Type
ID (unique)	long int	ID	long int
InitialDate	date	Day	date
FinalDate	date	geom	geometry
geom	geometry		

The initial date of each fire event is used as a criterion to assign the fire to the different yearly tables. The data is available in PANGAEA repository, data citation^26^.

## Technical Validation

### Inputs used from MCD64A1

The burnt area product chosen for this work was MCD64A1 produced using MODIS. The MCD64A1 is provided monthly in different formats tiled in 24 overlapping windows. Two of them are raster format using GeoTIFF or HDF5; the vector format is an ESRI Shapefile. However, only the raster format contains all the data generated during the processing for each pixel (such as burnt pixel uncertainty, date, quality) of the MCD64A1 product. The vector format is used in this case since the main goal is to get additional information which is not explicitly in MCD64A1, such as the individual fire event in addition to its initial and final date. When using the vector format provided for MCD64A1 the only additional information for feature is the burnt date. Detailed information about the burnt area product can be found in the user guide^19^ (http://modis-fire.umd.edu/files/MODIS_C6_BA_User_Guide_1.2.pdf).

### Resources used during the execution and database size

The data contained in each fire object is a set of daily burnt areas. Each object computes the final burnt area based on daily burnt areas, and fire dates are computed during the execution(s). For this first approach, only final areas, the daily burnt areas, time stamps and the fire number identification are saved in the PostgreSQL database.

This section shows the results and global statistics for a use case. Section 0.3 shows the fires which took place in Chile in 2017^27^. Next, section 0.4 presents statistics at global making use of the individual fire detection.

### Use case: chile fires 2017

**Fig. 2:**
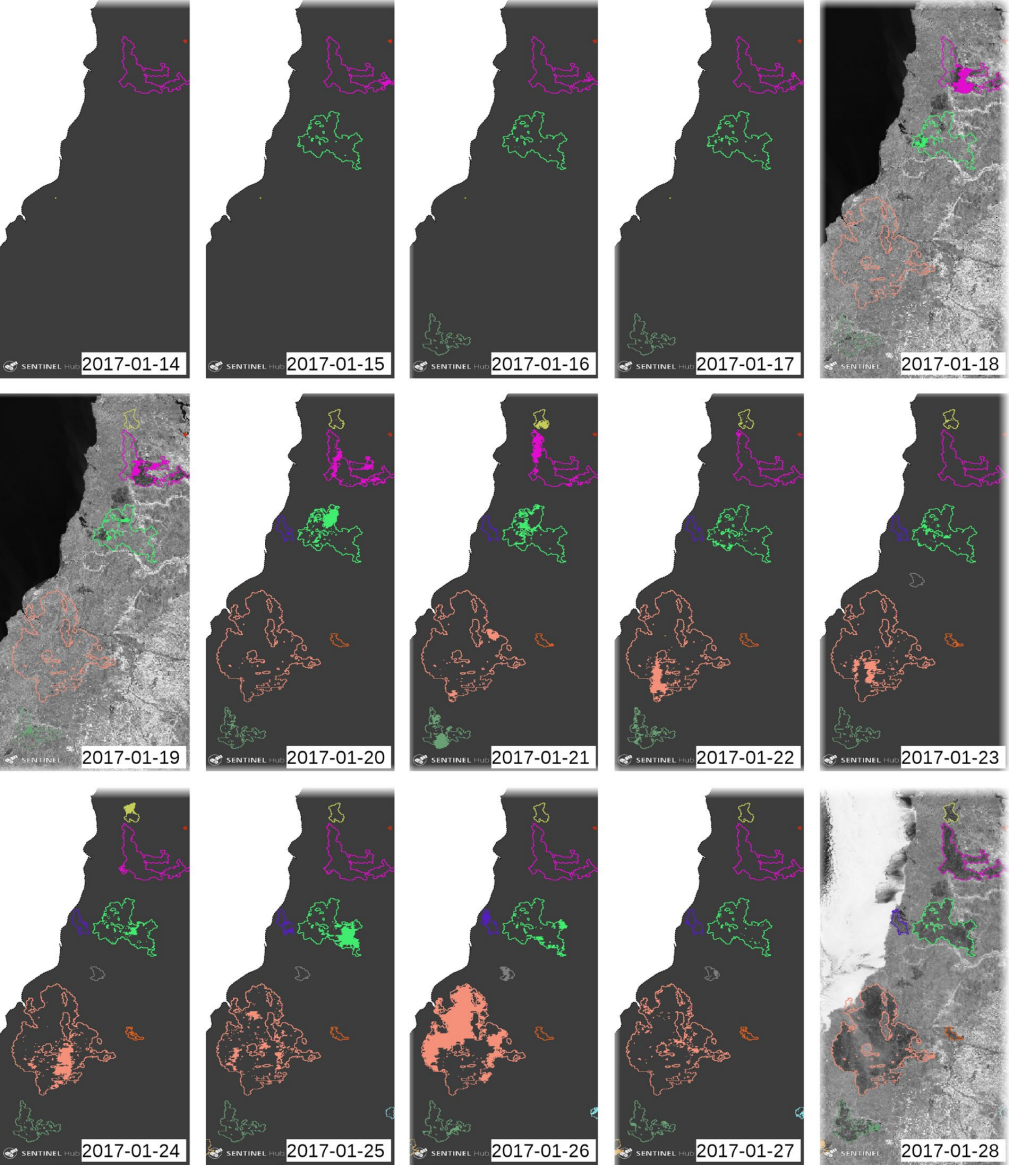
Active fire perimeters from 2017-01-14 to 2017-01-28 in Chile, near to Constitución area. Each final perimeter is shown, when active, with a different colour with respect to other fires. The daily burnt areas are shaded with the same colour of its perimeter. The background is the band 8 of Sentinel-2 when there is an image close in time (1 day distance).

Figure 2 depicts the the information that this work adds to the source. The colours are assigned randomly for each individual fire. The colour used for each fire event is based on the unique number identification and the final fire perimeters are shown only if they have daily burnt areas. The daily burnt areas are represented by shaded areas of the same colour as the final perimeter of each fire, since they have the same identification number. Therefore, it is crucial to identify every individual fire and to be able to analyse the daily burnt area, spread speed, etc. This fact makes it possible to distinguish between different fire types and to study their context.

### Statistics

Wildfire statistics usually report burnt areas or daily burnt areas, instead of number of fires, average fire sizes or spread speed. One of the reasons for this is the lack of databases and methods for counting wildfires and recording their evolution. Although, there are some organizations doing these tasks for specific areas, such as CALFIRE^28^ or EFFIS^29^.

Table 2 shows a comparison of large fires in California using the GlobFire database and the top 20 largest wildfires of CALFIRE(https://www.fire.ca.gov/media/5510/top20_acres.pdf). CALFIRE identifies each wildfire using the best available method to map the fire. All the biggest fires reported by CALFIRE from 2001 to 2017 were found in the GlobFire database. However, the GlobFire database also reports some additional big fires and the total burnt area of each fire is considerably different. Although both lists of large fires are similar, there are differences which may be caused by the difference of the human interpretation of the fire event against the automatic method of GlobFire and the different sources and methods used to compute the burnt area. Therefore, GlobFire is suitable for querying data about extreme fires in California.

**Table 2:** Comparison between the top 20 largest fires published by CALFIRE and the output of a GlobFire query for California, sorting by fire size. Burnt area unit is acres keeping the original data of table^28^.

CALFIRE		GlobFire			
Name	Area	Id	initialdate	finaldate	area
Biscuit Fire		1491299	2002-07-15	2002-09-03	421784.41
RUSH	315577	14533548	2012-08-12	2012-08-25	283879.60
CEDAR	273246	3281952	2003-10-22	2003-11-07	267823.67
THOMAS	281893	20855568	2017-12-01	2017-12-24	267722.77
RIM	257314	15706460	2013-08-15	2013-09-19	217156.73
ZACA	240207	8137811	2007-07-28	2007-08-27	206678.38
WITCH	197990	8600612	2007-10-15	2007-10-31	160780.34
STATION	160557	10864951	2009-08-26	2009-09-08	160718.39
DAY FIRE	162702	7119555	2006-09-04	2006-10-06	157459.58
BASIN COMP.	162818	9348287	2008-06-19	2008-08-02	154612.38
		3281549	2003-10-20	2003-11-09	140728.31
McNALLY	150696	1491909	2002-07-19	2002-08-20	133653.93
ROUGH	151623	18030161	2015-08-04	2015-09-25	125895.76
		19164360	2016-07-22	2016-09-17	112639.39
HAPPY CAMP	134056	16844497	2014-08-11	2014-09-21	100248.45
		8600652	2007-10-15	2007-10-31	92929.19
		3281505	2003-10-22	2003-11-05	92307.16
		10864905	2009-08-09	2009-08-23	88610.98
		17895908	2015-07-27	2015-08-17	85738.45

**Fig. 3:**
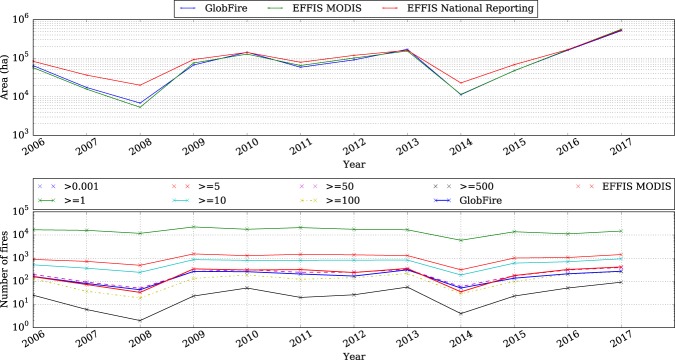
Annual burnt area and number of fires for EFFIS MODIS and GlobFire. The EFFIS national reporting is shown with different lines using a minimum threshold for the fire size shown in the legend (number of fires bigger than a number of size threshold).

Another potential source to compare GlobFire to is the current EFFIS database for European countries. For this comparison Portugal has been chosen for the comparison due to the low proportion of agricultural fires. EFFIS strictly maps forest fires excluding agricultural fires. GlobFire database does not filter by land cover. Therefore, an area with a known proportion of agricultural fires is better for a comparison of the results of this work. EFFIS has two databases: one uses MODIS for fire mapping^30,31^ and the other one is based on the reports provided by countries^32^. The one based on the reports has been used to compute the percentage of agricultural fires per year. The comparison of the three databases can be seen in Fig. 3. Considering the magnitude of the burnt area in Portugal, the agreement of the three different sources is strong. Besides, there is no relation between the percentage of agricultural fires and the agreement of the databases.

Considering that the main contribution of this work is fire event recognition, a comparison of the fire counts using EFFIS and GlobFire was carried out. Using the national reports from Portugal we can quantify the fire counting by different minimum fire sizes and compare it with the fire counting produced by EFFIS remote sensing and GlobFire. Figure 3 (below) shows the number of fires reported by Portugal differentiated by minimum fire size. The number of fires provided by EFFIS from remote sensing and the number of fires recognised by GlobFire are also in Fig. 3 allowing us to determine which is the minimum fire size seen by EFFIS remote sensing and GlobFire. In the figure, GlobFire is between two lines of the national reporting using a different line style, one with continuous line style in green(number of fires bigger or equal to 10 ha) and the other one dotted in yellow(number of fires bigger or equal than 100 ha). These lines show that GlobFire and EFFIS identify fires with a minimum size between 10 and 100 ha. Besides, there is a difference between the fire definition of a fire event between GlobFire and EFFIS. For EFFIS, the same burnt area which has been generated from two different ignition sources is considered as two different fires. However GlobFire criteria differs from EFFIS, considering the same situation as a single fire if both fires are still active (5 days threshold) when the burnt areas touch each other.

**Fig. 4:**
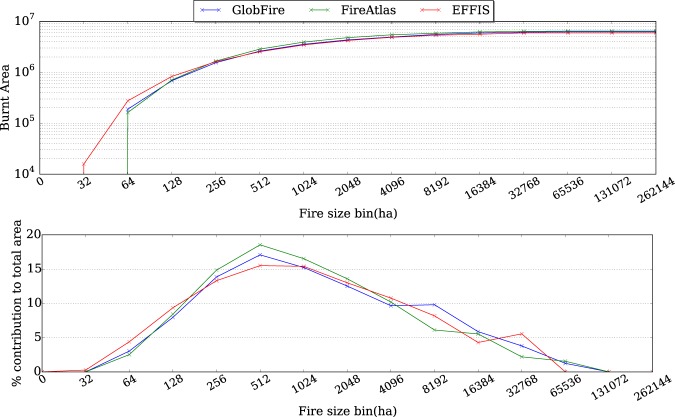
Accumulated burnt area and percentage of contribution to the total burnt area depending on the fire size for Europe extent.

Figure 4 (above) shows the accumulated burnt area from EFFIS, Fire Atlas and GlobFire. Fire Atlas and GlobFire match quite well on the total amount of burnt area. EFFIS burnt area, based on remote sensing, relies on a human supervised method, while Fire Atlas and GlobFire have been filtered to remove agricultural fires using CCI Land Cover^33^ using the same criteria. The criteria used for this automatic filtering implies that a fire should have at least 10% of the total burnt area in non agricultural vegetation and the amount of burnt area of non agricultural vegetation is more than 100 ha. Figure 4 (below) depicts the contribution to the total burnt area depending on the fire size. Although EFFIS also shares the fact that one wildfire has a single fire ignition, Fire Atlas splits the wildfires more than EFFIS, except for fire sizes between 8192 and 16384 ha. GlobFire merges the fire events showing a higher proportion than the other sources from 4096 to 65536 ha final fire sizes.

**Fig. 5:**
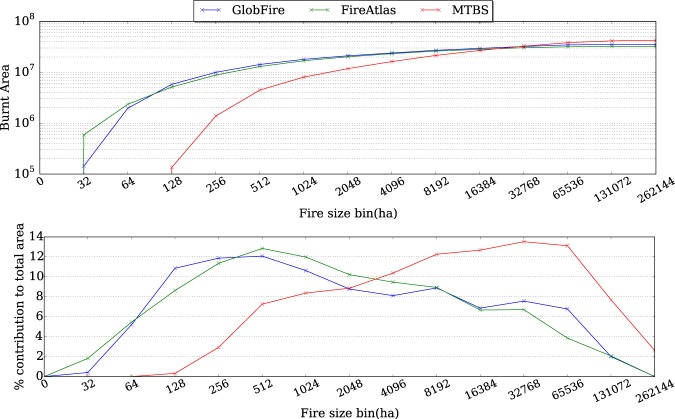
Accumulated burnt area and percentage of contribution to the total burnt area depending on the fire size for USA extent.

A similar comparison has been carried out using the Monitoring Trends in Burn Severity (MTBS), for USA, filtering agricultural fires with the same criteria as in the EFFIS comparison. Figure 5 above shows that MTBS presents a considerable difference caused by the fact that the minimum fire size is 500 and 1000 acres (202.343 and 404.686 ha), at the Eastern US and Western US, respectively. Then, the contribution to the total burnt area depending on the fire size is considerably different, see Fig. 5 below. GlobFire and Fire Atlas have, in general, a similar behaviour, but Fire Atlas splits large fires (more than 16384 ha), increasing the contribution of fires from 512 ha to 4096 ha. Despite the difference between MTBS with respect to the matching trend of FireAtlas and GlobFires, the trend (not the magnitude) of contribution of those fires bigger than 16384 ha is quite similar between MTBS and FireAtlas.

However, Fig. 5 (below) also shows a considerable agreement of GlobFire with Fire Atlas, MTBS the one with largest contribution of bigger fires. It is worth mentioning that, Fire Atlas and GlobFire database, show a big difference for fires bigger than 8192 ha. Fire Atlas splits these fires having a considerable contribution of fires between 512 ha and 4096 ha.

In both comparisons, for Europe and USA, the extent and the temporal window used for FireAtlas and GlobFire has been defined by the reference data (EFFIS and MTBS).

**Fig. 6:**
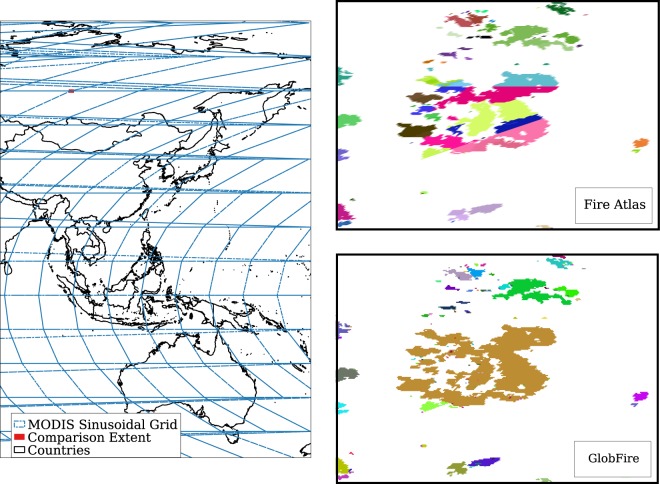
The figure on the left shows an area of the world overlapping the 10° × 10° MODIS sinusoidal grid and in red the extent of comparison used in the two images in the right column. The figures in the right column show a zoomed area, applying random colours for each fire event of 2016 to make it easier to distinguish between them. Fire Atlas data of 2016 shows the consequences of using fire event identification at tile level producing an artificial splitting of the fires which follows the pattern of the MODIS tile grid. GlobFire shows the same area of interest for the same year 2016 using GlobFire without any tile pattern.

This difference between the Fire Atlas and the GlobFire database relies on the difference in criteria of one initial ignition per fire and the tile and source independent method used in GlobFire. Figure 6 illustrates the reason why it is important to use tile independent methods for wildfire event detection. The centre image shows a visible vertical and horizontal artifact since the identification is carried out by tiles in the Fire Atlas. Those fires crossing boundary tiles are split and detected as different fires for each tile. Therefore, any posterior analysis on areas affected by tile boundaries is biased if the wildfire event method is not tile independent.

An assessment performed in Mato Grosso (Brazil)^34^ compared an early version of the GlobFire database with the Tropical Ecosystems and Environmental Sciences (TREES) burned area product, a manual edition of burnt polygons based on MODIS/Terra (with higher spatial resolution than the one of GlobFire). The comparison found a similar frequency distribution of the polygon area (with a difference respectively of 9% in total burnt area, and not greater than 11% for medium and large polygons), with the only significant difference in area found for smaller polygons (21%).

**Fig. 7:**
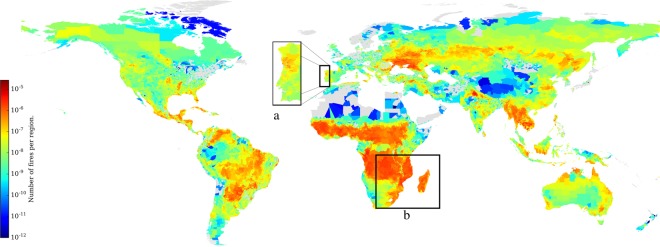
Number of fires per region surface from 2001 to 2017.

The GlobFire database has been used to determine the number of fires, the average fire size and the maximum fire spread speed for each administrative 3rd level region from 2001 to 2017. Those countries without 3rd administrative level in the Global Administrative Areas(GADM)(https://gadm.org) have been ignored. For instance, see Fig. 7 for the fire density. The change is quite smooth between regions for some zones. However, there are some close regions with a considerable difference between their values. GlobFire can be used for several kinds of analysis. In this work, a general overview, including some fire behaviour parameters, is summarised at the 3rd level region.

**Fig. 8:**
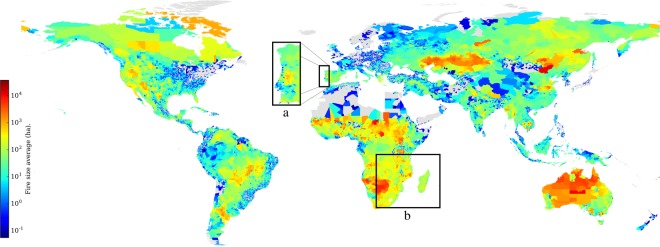
Fire size average per region 2001 to 2017.

Figure 7 shows the fire density. This information identifies area with a high number of fires of a moderate average fire size (Fig. 8). For instance, focusing on Portugal, the number of fires is considerable higher in the north of the country. However, the average fire size is much higher in the central area of Portugal. See annotation a in Fig. 8. Then, it can be concluded that the fires, on average, are bigger in the central part than in the northern area of Portugal. A similar situation is shown when comparing Madagascar with eastern Mozambique, annotation b. In this last case, it can be concluded that fires in Madagascar are more sparse than in some areas of eastern Mozambique like around the Gilé National Reserve.

Since the fires are individually located, some basic fire properties can be analysed for the events affecting each region. Recently, several works^35–39^ establish the links between climate change and fires. In addition the fire fighting community noted that fires are getting more intense and harder to fight^40^.

**Fig. 9:**
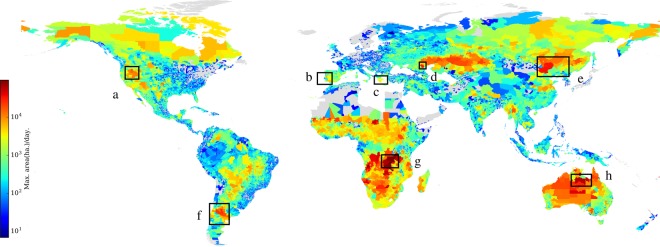
Max. fire average fire spread per region from 2001 to 2017.

Figure 9 shows the maximum fire spread speed of those fires which affected a region. In Europe, the fastest moving fires occur in south of Spain (b), in the Peloponnese (Greece, annotation c) and specially in central Portugal (b). From the global perspective, Santa Isabel (Argentina, annotation f), Malheur (USA, annotation a), Tableland (Australia, annotation h), Halhgol (Mongolia, annotation e), Chernozermel’skiy rayon (Russia, annotation d) and the Republic of Tanganika (g) have the fastests moving fires. Locations of the regions with fastest fire spread propagation match with the regions where wildfires were most unpredictable and harmful. In Europe, the central part of Portugal, south Spain and Greece stands out from the rest of Europe and matches with the most tragic fire events for this continent.

## Usage Notes

The data generated on this work is accessible from the mentioned repository and also in the GWIS. It may be convenient for the potential users who want to do studies of areas of interest to request the data directly from GWIS. For the users who want to use the data for global studies it is recommended to use the PostgreSQL dump files containing the final fire perimeters. In addition, the daily burnt areas are also provided for those users who want to study the cases by their daily evolution.

## Data Availability

The code is available on demand from the EFFIS or GWIS team of the Joint Research Centre of the European Commission. Two parameters have been used in the execution of the application to the MCD64A1. First, the fire is considered as ended when it has been 16 days without activity. The second parameter is the time between two burnt areas that touch each other: if one of them has been active in the last 5 days it will become a single fire event. The code developed in GWIS uses the previously mentioned libraries and processes a single delivery of a burnt area product. The spatio-temporal clustering is defined during the creation process of the sparse matrix.
